# A human iPSC‐derived neurosphere model to study functional and transcriptomic properties of microglial neuroprotection in AD

**DOI:** 10.1002/alz70855_098607

**Published:** 2025-12-23

**Authors:** Stefan Wendt, Ada J Lin, Sarah N Ebert, Declan J Brennan, Wenji Cai, Yanyang Bai, Da Young Kong, Stefano Sorrentino, Christopher J Groten, Christopher Lee, Jonathan Frew, Hyun B Choi, Konstantina Karamboulas, Mathias Delhaye, David R Kaplan, Freda Miller, Brian A MacVicar, Haakon B. Nygaard

**Affiliations:** ^1^ University of British Columbia, Vancouver, BC, Canada; ^2^ Opalia, Montreal, QC, Canada; ^3^ UBC Hospital Clinic for Alzheimer Disease and Related Disorders, Vancouver, BC, Canada

## Abstract

**Background:**

Alzheimer's disease is characterized by the accumulation of Aβ plaques in the brain, but the pathways leading from early Aβ deposits to eventual neurodegeneration are not fully understood. The specific role of microglia in AD is controversial, with microglial activation to be protective or detrimental depending on disease progression. We developed a 3D human iPSC‐derived neurosphere model to better characterize how microglia respond to Aβ pathology.

**Method:**

Neurospheres were grown from iPSC‐derived neuronal progenitor cells and cultured for 60 days. iPSC‐derived microglia were added separately to assess their functional impact. Continuous synthetic oligomeric Aβ treatment was used to model amyloidosis. Genetically encoded sensors roGFP1 and GCaMP6f allowed to monitor neuronal oxidative stress and calcium activity, respectively. Single nuclei RNA sequencing (snRNA‐seq) was performed to examine transcriptomic differences induced by Aβ and microglia.

**Result:**

Neurospheres grew to consist of a complex network of healthy mature neurons and astrocytes. The added microglia readily infiltrate the 3D tissue. Chronic Aβ treatment resulted in the formation of plaque‐like aggregates and subsequently led to severe oxidative stress and loss of neuronal activity. Microglia were able to prevent these neurotoxic effects in neurospheres that received mild 3 weeks Aβ treatment, but failed to do so after severe 5 weeks treatment. snRNA‐seq revealed that, after 3 weeks treatment, microglia induced upregulation of genes linked to oxidative regulation in astrocytes. After 5 week treatment, microglia more noticeably drove the expression of many AD‐associated genes, such as APOE, CLU, and FTL, in astrocytes and neurons. Microglia also appear to have enhanced ability for Aβ clearance after treatment with anti‐Aβ antibodies.

**Conclusion:**

We developed a human 3D neurosphere model to study microglial responses to Aβ pathology. In this model, microglia acquire a neuroprotective phenotype which may be enhanced by anti‐Aβ antibodies. Microglia are essential for driving the transcriptomic profile linked to AD after severe Aβ treatment. By mimicking critical AD pathological features, this model offers a platform to identify therapeutic targets with translational relevance.

